# INTIMA-MEDIA THICKNESS OF THE CAROTID ARTERIES IS AFFECTED BY PUBERTAL MATURATION IN HEALTHY ADOLESCENTS

**DOI:** 10.1590/1984-0462/;2019;37;4;00010

**Published:** 2019-07-04

**Authors:** Jovita Lane Soares Santos Zanini, Tânia Maria Barreto Rodrigues, Cristina Botelho Barra, Maria Francisca Tereza Freire Filgueiras, Ivani Novato Silva

**Affiliations:** a Universidade Federal de Minas Gerais, Belo Horizonte, MG, Brazil.; b Universidade Federal de Juiz de Fora, Valadares, MG, Brazil.

**Keywords:** Ultrasonography, Risk factors, Carotid intima-media thickness, Adolescents, Carotid arteries, Ultrassonografia, Fatores de risco, Espessura íntima-média carotídea, Adolescentes, Artérias carótidas

## Abstract

**Objective:**

To contribute to the assessment of normal parameters of carotid intima-media thickness (CIMT) in healthy adolescents.

**Methods:**

A cross-sectional study was conducted through clinical, laboratory and ultrasound evaluation in 61 healthy adolescents. The inclusion criteria consisted of being in good health. The exclusion criteria were: presence or history of any chronic disease; being obese or overweight according to the World Health Organization (WHO) established criterion; continuous use of medication; or presenting a febrile condition or requiring medication within 48-hours prior to assessment. The pubertal stages were evaluated using the Tanner criteria. The high-resolution B-mode ultrasound examinations were performed according to the recommendations of the Consensus Statement from the American Society of Echocardiography Carotid Intima-Media Thickness Task Force.

**Results:**

Adolescents were 14±2.6 years old, 62.3% female, 19 (31%) at early puberty (Tanner II and III), and 38 (62%) at late puberty (Tanner IV and V). They presented normal clinical and laboratorial parameters. CIMT values were 0.46±0.04 to 0.55±0.04 mm on the right and 0.48±0.02 to 0.53±0.04 mm on the left, according to pubertal maturation. CIMT values increased significantly on the right and left sides, according to pubertal stage (p<0.001 and p=0.016), and maximum internal diameters of the common carotid artery (p<0.025 and p<0.003). It was higher in males compared to females.

**Conclusions:**

An increase in CIMT in the healthy adolescents group, according to both age, and the degree of pubertal maturation should be considered when evaluating adolescents in diagnostic procedures.

## RESUMO

**Objetivo:**

Contribuir para a avaliação dos parâmetros normais da espessura médio-intimal carotídea (EMIC) em adolescentes saudáveis.

**Métodos:**

Estudo transversal realizado por meio de avaliações clínicas, laboratoriais e ultrassonográficas em 61 adolescentes saudáveis. O critério de inclusão foi ter boa saúde. Os critérios de exclusão foram: presença ou histórico de doença crônica; obesidade ou sobrepeso segundo os parâmetros estabelecidos pela Organização Mundial da Saúde (OMS); uso contínuo de medicação; e quadro febril ou que necessitasse de uso de medicação nas 48 horas anteriores à avaliação. Os estágios puberais foram avaliados pela escala de Tanner. As ultrassonografias em modo B de alta resolução foram realizadas seguindo as recomendações do *Consensus Statement from the American Society of Echocardiography Carotid Intima-Media Thickness Task Force (*Declaração de Consenso da Força Tarefa da Sociedade Americana de Ecocardiografia sobre Espessura Médio-Intimal Carotídea).

**Resultados:**

Os adolescentes tinham 14±2,6 anos, 62,3% eram do sexo feminino, 19 (31%) estavam em estágios iniciais da puberdade (2 e 3) e 38 (62%) em estágios avançados (4 e 5) de acordo com a escala de Tanner. Todos apresentavam parâmetros clínicos e laboratoriais normais. Os valores da EMIC variaram de 0,46±0,04 a 0,55±0,04 mm do lado direito e 0,48±0,02 a 0,53±0,04 mm do lado esquerdo, conforme a maturação puberal. Houve aumento significativo nos valores da EMIC em ambos os lados de acordo com o estágio puberal (p<0,001 e p=0,016) e os diâmetros internos máximos da artéria carótida comum (p<0,025 e p<0,003). A EMIC foi maior em participantes do sexo masculino em relação ao feminino.

**Conclusões:**

O aumento da EMIC em adolescentes saudáveis, conforme a idade e o grau de maturação puberal, deve ser levado em consideração nas avaliações diagnósticas.

## INTRODUCTION

The carotid intima-media thickness (CIMT) has become a standard surrogate marker of early vascular changes, and has been widely used in adults.^1^ ^,^ ^2^ In pediatric cohorts, it has been also associated with cardiovascular risk factors, such as a family history of hypercholesterolemia, obesity, hypertension, and diabetes.^3^ ^,^ ^4^ ^,^ ^5^ ^,^ ^6^ ^,^ ^7^

Evidence for incipient atherosclerosis was found at very early ages suggesting that the pathophysiological basis of this disease begins in childhood, and that cardiovascular disease (CVD) could be prevented in some situations.^8^ ^,^ ^9^

Besides the adoption of a healthy lifestyle, considered essential to reduce the impact of CVD on global morbidity and mortality, other strategies could be developed to modify the vascular profile after the identification of early cardiometabolic markers in clinical practice.^10^

It has been proposed that the measurement of CIMT should be introduced as a tool for detection and monitoring of target-organ damage in children with cardiovascular risk factors.^11^ ^,^ ^12^

However, like other measurements of biological variables, a clear definition between physiological and pathological conditions in the pediatric population is needed. There are few reports in the literature focusing on the distribution and determinants of CIMT in healthy adolescents and doubts remain on the factors that influence carotid thickening.^13^ ^,^ ^14^ ^,^ ^15^

The present study aims to contribute to the assessment of normal parameters of the CIMT in healthy adolescents, using high-resolution ultrasound (US).

## METHOD

This cross-sectional study was approved by the Institutional Review Board (ETIC 302/08) of the Universidade Federal de Minas Gerais, Belo Horizonte, Brazil, and the consent of the institutions involved was obtained. To be included, all adolescents and legal guardians signed a written Informed Consent Form after receiving information concerning the study.

Healthy adolescents from both genders (10-20 years) were randomly recruited among students of a public school located in the central area of the city. The inclusion criteria consisted of being in good health. A cohort of 80 adolescents was evaluated by the same pediatric endocrinologist at the University Hospital (HC-UFMG). A complete physical examination was conducted. The exclusion criteria were: presence or history of any chronic disease; being obese or overweight according to the World Health Organization (WHO) established criterion (body mass index for age and gender above the 85^th^ percentile);^16^ continuous use of medication; or presenting a febrile condition or requiring medication within 48-hours prior to assessment.

The anthropometric parameters assessed according to the WHO criteria were measured using digital scales (G-Tech^®^) to the nearest 0.1 kg for weight, and a wall-mounted stadiometer to the nearest 0.1 cm for height. The body mass index (BMI) Z scores were classified according to the WHO established criteria.

Pubertal staging for female breast development and for male genitalia were evaluated using the Tanner criteria.^17^ To analyze the pubertal stages, the participants were classified as prepubescent (Tanner 1), early puberty (Tanner 2 and 3), and late puberty (Tanner 4 and 5).

Blood pressure was measured with the subjects in the supine position, using a calibrated, age-specific sphygmomanometer (Tycos, Welch Allyn, New York, United States), and analyzed following The Fourth Report on the Diagnosis, Evaluation, and Treatment of High Blood Pressure in Children and Adolescents, according to age and height.^11^

Following overnight fasting, blood samples were collected between 8 and 9 a.m., at the HC-UFMG Laboratory, for the biochemical assessment. Serum glucose, total cholesterol and fractions, and triglycerides were measured by colorimetric methods (Vitros® 5.1 FS Chemistry System, Buckinghamshire, United Kingdom). The serum lipid concentrations were evaluated according to the First Guidelines for Prevention of Atherosclerosis in Childhood and Adolescence of the Brazilian Society of Cardiology (reference values - RV: total cholesterol - CT<170; low-density lipoprotein - LDL-C<130; triglicerides - TG<130 mg/dL),^18^ and glucose levels were considered as per the recommendations from the American Diabetes Association (RV<100 mg/dL).^19^

After the clinical and biochemical evaluations, 19 subjects were excluded, and a final sample was composed of 61 adolescents.

High-resolution B-mode ultrasonography was performed by a single, trained examiner, a specialist in radiology and diagnostic imaging, to evaluate the measurements of the intima-media thickness (IMT) of the carotid arteries, based on the recommendations of the Consensus Statement from the American Society of Echocardiography Carotid Intima-Media Thickness Task Force.^2^ The examinations were carried out using a Philips device, model HD11XE, equipped with a linear, high-resolution, multi-frequency transducer set to 12 MHz. Photographic documentation of the B-mode was done using the image acquisition program Image Explorer 3.1 for Windows. After resting for 10 minutes, the patients were evaluated in the supine position with the neck slightly extended and inclined to 45º. Longitudinal and transverse sections of the right and left common carotid arteries (CCA), bulbs, and internal and external carotid arteries were obtained for combined ultrasound evaluations using B-mode.

The analysis was carried out focusing on the identification of the intima-media layer and finding of focal atherosclerotic plaques. The examiner measured the maximum internal diameters and the CIMT of the common carotid arteries. Themeasurements were manually performed on the posterior wall, the farthest from the transducer using the device calibrator. Three measurements of IMT of the right and left common carotid arteries were obtained in the 15-mm stretch below the bulb region, free from focal plaques, in which the double-line pattern of IMT can be clearly observed, to enable the analysis of the mean values of each artery.

Descriptive statistics were performed, and data are presented as measurements of central tendency and dispersion (median and mean±standard deviation) for continuous variables, and as proportions for categorical and quantitative variables. Normal distribution of the variables was tested using the Shapiro-Wilk test for normality and Levene test for homoscedasticity (homogeneity among the variances).

Comparisons between the response variables and binary covariates were made using the Student’s *t* test. Comparisons of variables with more than two categories were made by means of the F test (ANOVA). The Pearson correlation coefficients (r) between the response variables and quantitative covariates were calculated.

Linear regression models were developed for the multivariate analysis. In the first step, all covariates with p≤0.25 in the univariate analysis were included: age, gender, Tanner stage, height, weight, BMI, and CCA diameter. The response variables were right and left carotid IMT. Next, the variables were removed in a stepwise process to the point that only the variables with statistical (p≤0.05) and clinical significance were included in the final model. The residue analysis was performed to verify adjustment adequacy.

At the beginning of the study, the inter-observer variability was evaluated by comparing the results of five randomly selected patients, who were examined by another experienced sonographer, blinded to the characteristics of the adolescent sample, to test reliability of CIMT evaluations. A high correlation index (intra-class correlation coefficient - ICC) between the researcher and the experienced sonographer was observed in the ultrasound evaluation (ICC=0.98 for right mean IMT and 0.95 for left mean IMT).

The statistical packages R 2.7.1 and Epi Info 6.04 were used for analysis. Rejection of the null hypothesis was set at 5% (p<0.05).

## RESULTS

Sixty-one healthy adolescents aged 14±2.6 years old (10.1-19.3) made up the reference sample group. There were 23 (37.7%) males and 38 (62.3%) females; 19 (31%) at early puberty (Tanner II and III), 38 (62%) at late puberty (Tanner IV and V), and 4 prepubertal. The degree of sexual maturity was similar in both genders (p=0.108), and the data from males and females were analyzed together.

There were no smokers among the adolescents, and eight (13.1%) reported social consumption of alcohol. The blood pressure, blood glucose and lipid levels were within the normal ranges for their ages. The clinical and laboratory characteristics of the adolescents are shown in Table 1.

**Table 1:** Clinical and laboratory characteristics of the 61 healthy adolescents from the study.

Characteristics	Mean	SD
Age (years)	14.3	2.6
Z score height/age	-0.01	1.0
Z score weight/age	-0.32	0.9
Z score BMI	-0.40	0.9
Tanner stages	Male (n)	Female (n)
1	4	0
2	2	6
3	3	8
4	5	11
5	9	13
Biochemical tests (mg/mL)	Mean	SD
Glucose	83.8	5.7
Triglycerides	72.0	26.8
Total cholesterol	148.5	19.1
HDL-cholesterol	49.6	8.4
LDL-cholesterol	84.7	17.8
VLDL-cholesterol	14.0	5.4
Systolic blood pressure (mmHg)	110.1	8.5
Diastolic blood pressure (mmHg)	72.2	7.2

BMI: body mass index; HDL: high density lipoprotein; LDL: low density lipoprotein; VLDL: lipoprotein; SD: standard deviation.

CIMT measurements and CCA maximum internal diameter of the 61 healthy adolescents are shown on Table 2. An increase in the diameter of CCA was associated with greater CIMT on both sides (right side: r=0.400, p<0.003; left side: r=0.305, p<0.025). A gender difference in CIMT measurements was observed, with significantly higher values in males (p<0.004), bilaterally (Table 2).

**Table 2:** Intima-media thickness and maximum internal diameter measurements (mean±standard deviation) of the carotid artery in the 61 healthy adolescents from the study.

	Right	Left
IMT (mm)	0.51±0.05	0.51±0.04
IMT female (mm)*	0.49±0.04	0.50±0.03
IMT male (mm)*	0.53±0.05	0.53±0.04
Diameter (mm)**	5.42±0.53	5.32±0.42

IMT: Intima-media thickness; SD: standard deviation; *p<0.004; ^**^measurements at end of the diastole.

**Figure 1:**
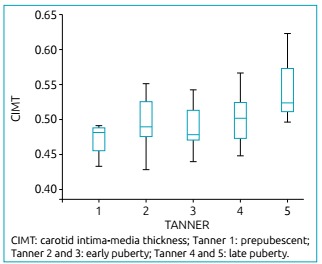
Association between pubertal development and carotid intima-media thickness (mm) in the 61 healthy adolescents from the study.

CIMT measurements were positively associated with the progression of pubertal maturation in both genders (Figure 1), and on both sides: r=0.561 for females and r=0.719 for males (p<0.001) on the right side, and r=0.541 for females and r=0.695 for males (p<0.001) on the left side.

CIMT measurements were also positively associated with pubertal Tanner stages. Adolescents presenting Tanner 5 showed the highest values in comparison to the others stages and to the prepuberal ones (Table 3).

**Table 3:** Distribution of carotid intima-media thickness according to Tanner stages in 61 healthy adolescents (mean±standard deviation).

Tanner stages	CIMT	
	Right	Left
M1 or G1*	0.46±0.04	0.48±0.02
M2 or G2	0.49±0.05	0.50±0.04
M3 or G3	0.48±0.03	0.50±0.04
M4 or G4	0.49±0.03	0.51±0.04
M5 or G5*	0.55±0.04	0.53±0.04

CIMT: carotid intima-media thickness; SD: standard deviation; *F test (ANOVA): right side p<0.001; left side p=0.016.

**Figure 2:**
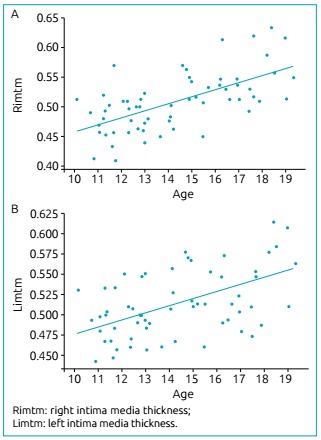
Association between age and carotid intima-media thickness in the 61 healthy adolescents from the study.

When comparing CIMT measurements according to age, there was positive association on both sides (r=0.619, p<0.001 on the right, and r=0.445, p<0.001 on the left side), and the highest values were observed among the oldest adolescents, as shown in Figure 2.

No significant association was found between CIMT and anthropometric parameters (height/age, weight/age or BMI/age Z scores) on either side.

For both sides, three regression models for the multivariate analysis were developed. For the left side, the first model included gender, Tanner stage, and weight; the second one included gender, Tanner stage, and BMI; and the third one, only the CCA diameter. According to the regression analysis, gender and pubertal stage, besides age, BMI, and CCA diameter influenced the CIMT values. For the right side, the first model included gender, and Tanner stage; the second one included age and weight; and the third one, age and the CCA diameter. According to the regression analysis, gender and pubertal stage, besides age, weight, and CCA diameter influenced the CIMT values. A summary of multivariate regression analysis is presented in Table 4.

**Table 4:** Summary of multivariate regression analysis of carotid intima-media thickness and associated variables in the 61 healthy adolescents from the study.

	Right			Left		
	I	II	III	I	II	III
Gender (male)	0.05 (0.03-0.07)	-	-	0.04 (0.03-0.06)	0.04 (0.03-0.06)	-
Tanner stage	0.06 (0.02-0.10)	-	-	0.05 (0.01-0.09)	0.05 (0.01-0.10)	-
M2 or G2	0.05 (0.02-0.09)	-	-	0.04 (0.01-0.08)	0.05 (0.01-0.09)	-
M3 or G3	0.06 (0.03-0.10)	-	-	0.05 (0.02-0.09)	0.06 (0.02-0.10)	-
M4 or G4	0.11 (0.08-0.15)	-	-	0.08 (0.04-0.11)	0.08 (0.04-0.12)	-
M5 or G5	-	0.001 (0.0001-0.001)	0.001 (0.0005-0.0011)	-	-	-
Age (months)	-	0.002 (0.001-0.003)	-	-	-	-
Weight (kg)	-	-	-	0.01 (0.005-0.02)	-	-
Z score weight /age	-	-	-	-	0.01 (0.002-0.02)	-
Z score BMI	-	-	0.25 (0.07-0.43)	-	-	0.29 (0.04-0.53)
CCA diameter (mm)						

Data are expressed in OR (95% confidence interval); BMI: body mass index; -: not included; CCA: common carotid arteries.

## DISCUSSION

We observed increase in CIMT according to age and progression of puberty. The diameter of the CCA was also significantly associated with intima-media layer thickness in the present study. This finding in healthy adolescents of both genders most likely reflects normal physiological growth.

The evaluation of the CIMT and of the intraluminal diameter of the CCA in children aged 5 to 14 years old suggested that progressive thickening of the intima-media layer could partly reflect normal physiological growth.^20^ It was suggested that greater CIMT would be a consequence of the increased diameter of the artery and should be interpreted in relation to this diameter.^21^

Likewise, the increase in IMT observed in both CCA according to increasing age has been associated with normal somatic growth and is related, mainly, to the physiological rise in blood pressure.^20^ It was also observed that this finding would be more significant during adolescence, suggesting a relation with pubertal development.^13^ ^,^ ^22^ Up to now, we found no reported data of a healthy adolescent group that assessed IMT increment using a strict protocol in relation to pubertal development. A continuous and significant increase of IMT was reported in girls who were at the beginning of their puberty, while in boys from the onset of puberty IMT stagnation was observed. The increase of IMT in those pubertal girls was associated with the increase in body fat and could be related to the effect of estrogens. Tanner scores were not obtained in the study, a limitation that was considered by the authors.^23^ It was not taken into account the differences existing between boys and girls in the pubertal development, as girls present the early physical signs of puberty approximately two years before boys. Our findings suggest there is a continuous and significant increase of IMT in both, boys and girls, probably related to the physiological rise in sex hormones, acting from the onset of puberty.

In the largest published series, a cohort of children from 6 to 18 years old, it was suggested children might be assessed more accurately by height-normalized reference values.^15^ The body size differences during puberty are observed not only between boys and girls, but also among individuals of the same gender, being fast or slow maturers. Therefore, it seems CIMT should be assessed not only by height-normalized values, but also by the Tanner stage of puberty.

There is little information in the literature regarding the distribution of CIMT in each gender. Higher values of CIMT in male adolescents found in this study has already been reported.^15^ ^,^ ^23^

Differences in IMT of the carotid artery between the right and left sides of the body are observed in adults, and reference values were established for each side.^24^ Although higher values of CIMT are found on the left side in adults, this difference has not been observed in adolescents, who presented with similar values in both, the right and left sides, in agreement with the findings of the present study.

Pediatric markers of CVD risk are being reporting,^25^ and numerous studies are consistent when stating that CIMT evaluation contributes to the early identification of adolescents who are at higher cardiovascular risk. CIMT may be increased in children with chronic diseases, suggesting early vascular damage with greater cardiovascular risk in the future.^26^ The hemodynamic damage is considered the first and most important event that triggers the pathologic remodeling of the arterial wall, causing IMT.^27^ Large observational longitudinal studies, such as the Muscatine Study and the Bogalusa Heart Study, demonstrated the presence of hypercholesterolemia and diabetes during childhood, and adolescence is closely related to higher values of CIMT and cardiovascular events in adult life.^28^ ^,^ ^29^

It was reported ideal cardiovascular health metrics was inversely associated with CIMT, suggesting that ideal cardiovascular health reflects vascular health at the population level.^3^ A drawback to the wider use of CIMT measurements in adolescents has been the lack of standardized CIMT values in this age group. There are few reports in the literature focusing on the distribution and determinants of CIMT in healthy children and adolescents. Besides, a variety of criteria for normal children had been used in the reports and, in most of them, the clinical and metabolic characteristics of the patients were not fully described.

In a published series, 20% of the adolescents had the lipid and glycemic profiles evaluated.^13^ In a cohort of subjects, 6 to 17 years old, to determine the CIMT percentile curves specific for age and gender, besides personal questionnaires for evaluating whether subjects were free from illnesses, the blood pressure was measured.^23^ For normative data based on 135 healthy children and adolescents, the authors performed cardiac evaluation, and defined low risk by exclusion of cardiovascular anomalies through interview, clinical examination, and echocardiography.^14^

The environmental and behavioral changes observed in recent years have been resulted in an increased incidence of cardiometabolic risks such as obesity, and dyslipidemia in children and adolescent population, all over the world.^30^ It would be desirable to exclude these conditions prior to establishing reference values, a strength of the present study.

According to gender and age range, the CIMT values evaluated through B-mode in other report (CIMT=0.53±0.03 mm)^23^ were similar to those ones presented here, but lower values were reported for adolescents in another studies (CIMT=0.38 to 0.41 mm).^13^ ^,^ ^14^ ^,^ ^15^ However, the direction of effect on CIMT in the studies was the same in all cohorts.

The carotid ultrasound was well tolerated and easy to perform in the adolescents, and there is an excellent resolution of the images in the pediatric population. These characteristics and the standardization of the test technique with high reproducibility in obtaining the CIMT measurements point to a good quality of data in this study.

To the best of our knowledge, this is the first study that analyzed CIMT according to the well-established Tanner criteria for pubertal development. It is the largest sample reported in which all healthy adolescents underwent an extensive clinical and laboratory workup, and the first study that describes IMT of the carotid arteries in Brazilian adolescents.

A major limitation of this study was the sample size, that is not suitable for preparing reference curves. Nevertheless, it was possible to detect significant associations between CIMT and the variables studied, and the observed increase according to puberty progression. The CIMT parameters presented here can be useful to help establishing the role of this tool as a screening test in adolescents to identify risk factors for CVD.

In conclusion, CIMT measurements increased according to CCA diameter, age and progression of puberty in healthy adolescents. CIMT values are higher in male adolescents as compared to females. These findings should be considered when adolescents are evaluated for diagnostic procedures.

## References

[1] Lorenz MW, Markus HS, Bots ML, Rosvall M, Sitzer M (2007). Prediction of clinical cardiovascular events with carotid
intima-media thickness: a systematic review and
meta-analysis. Circulation.

[2] Stein JH, Korcarz CE, Hurst RT, Lonn E, Kendall CB, Mohler ER (2008). Use of carotid ultrasound to identify subclinical vascular
disease and evaluate cardiovascular disease risk: a consensus statement from
the American Society of Echocardiography Carotid Intima-Media Thickness Task
Force. Endorsed by the society for Vascular Medicine. J Am Soc Echocardiogr.

[3] Oikonen M, Laitinen TT, Magnussen CG, Steinberger J, Sinaiko AR, Dwyer T (2013). Ideal cardiovascular health in young adult populations from the
United States, Finland, and Australia and its association with cIMT: the
International Childhood Cardiovascular Cohort Consortium. J Am Heart Assoc.

[4] Kusters DM, Wiegman A, Kastelein JJ, Hutten BA (2014). Carotid intima-media thickness in children with familial
hypercholesterolemia. Cir Res.

[5] Geerts CC, Evelein AM, Bots ML, van der Ent CK, Grobbee DE, Uiterwaal CS (2012). Body fat distribution and early arterial changes in healthy
5-year-old children. Ann Med.

[6] Sorof JM, Alexandrov AV, Garami Z, Turner JL, Grafe RE, Lai D (2003). Carotid ultrasonography for detection of vascular abnormalities
in hypertensive children. Pediatr Nephrol.

[7] Fusaro MF, Zanini JL, Silva IN (2016). Increased carotid intima-media thickness in Brazilian adolescents
with type 1 diabetes mellitus. Diabetol Metab Syndr.

[8] Barker DJ, Osmond C, Golding J, Kuh D, Wadsworth ME (1989). Growth in utero, blood pressure in childhood and adult life, and
mortality from cardiovascular disease. BMJ.

[9] Järvisalo MJ, Jartti L, Näntö-Salonen K, Irjala K, Rönnemaa T, Hartiala JJ (2001). Increased aortic intima-media thickness: a marker of preclinical
atherosclerosis in high-risk children. Circulation.

[10] Roth GA, Huffman Moran AE, Feigin V, Mensah GA, Naghavi M (2015). Global and regional patterns in cardiovascular mortality from
1990 to 2013. Circulation.

[11] National High Blood Pressure Education Program Working Group on High
Blood pressure in Children and Adolescents (2004). The fourth report on the diagnosis, evaluation, and treatment of
high blood pressure in children and adolescents. Pediatrics.

[12] Barton M, US Preventive Services Task Force (2010). Screening for obesity in children and adolescents: US Preventive
Services Task Force recommendation statement. Pediatrics.

[13] Jourdan C, Wühl E, Litwin M, Fahr K, Trelewicz J, Jobs K (2005). Normative values for intima-media thickness and distensibility of
large arteries in healthy adolescents. J Hypertens.

[14] Sarkola T, Manlhiot C, Slorach C, Bradley TJ, Hui W, Mertens L, at al (2012). Evolution of the arterial structure and function from infancy to
adolescence is related to anthropometric and blood pressure
changes. Arterioscler Thromb Vasc Biol.

[15] Doyon A, Kracht D, Bayazit AK, Deveci M, Duzova A, Krmar RT (2013). Carotid artery intima-media thickness and distensibility in
children and adolescents: reference values and role of body
dimensions. Hypertension.

[16] World Health Organization (2009). AnthroPlus for personal computers manual: software for assessing growth
of the world's children and adolescents.

[17] Tanner JM, Forfar JO, Arnell CC (1978). Physical growth and development. Nelson textbook of pediatrics.

[18] Back Giuliano IC, Caramelli B, Pellanda L, Duncan B, Mattos S, Fonseca FH (2005). I Guideline for Prevention of Atherosclerosis in Childhood and
Adolescence. Arq Bras Cardiol.

[19] American Diabetes Association (2015). Standards of Medical Care in Diabetes 2015. Diabetes Care.

[20] Ishizu T, Ishimitsu T, Yanagi H, Seo Y, Obara K, Moriyama N (2004). Effect of age on carotid arterial intima-media thickness in
childhood. Heart Vessels.

[21] Yan RT, Anderson TJ, Charbonneau F, Title L, Verma S, Lonn E (2005). Relationship between carotid artery intima-media thickness and
brachial artery flow-mediated dilation in middle-aged healthy
men. J Am Coll Cardiol.

[22] Raitakari OT, Juonala M, Kähönen M, Taittonen L, Laitinen T, Mäki-Torkko N (2003). Cardiovascular risk factors in childhood and carotid artery
intima-media thickness in adulthood: the Cardiovascular Risk in Young Finns
Study. JAMA.

[23] Böhm B, Hartmann K, Buck M, Oberhoffer R (2009). Sex differences of carotid intima-media thickness in healthy
children and adolescents. Atherosclerosis.

[24] Luo X, Yang Y, Cao T, Li Z (2011). Differences in left and right carotid intima-media thickness and
the associated risk factors. Clin Radiol.

[25] Olson M, Chambers M, Shaibi G (2017). Pediatric markers of adult cardiovascular disease. Curr Pediatr Rev.

[26] Lamotte C, Iliescu C, Libersa C, Gottrand F (2011). Increased intima-media thickness of the carotid artery in
childhood: a systematic review of observational studies. Eur J Pediatr.

[27] McMahan CA, Gidding SS, Viikari JS, Juonala M, Kahonen M, Hutri-Kahonen N (2007). Association of pathobiologic determinants of atherosclerosis in
youth risk score and 15-year change in risk score with carotid artery
intima-media thickness in young adults (from the cardiovascular risk in
young finns study). Am J Cardiol.

[28] Davis PH, Dawson JD, Riley WA, Lauer RM (2001). Carotid intima-medial thickness is related to cardiovascular risk
factors measured from childhood through middle age: the Muscatine
study. Circulation.

[29] Berenson GS, Srinivasan SR, Bao W, Newman WP, Tracy RE, Wattigney WA (1998). Association between multiple cardiovascular risk factors and
atherosclerosis in children and young adults. The Bogalusa Heart
Study. N Engl J Med.

[30] Skinner AC, Perrin EM, Moss LA, Skelton JA (2015). Cardiometabolic risks and severity of obesity in children and
young adults. N Engl J Med.

